# Sinonasal and Skull Base Metastatic Renal Cell Carcinoma: A Case Series

**DOI:** 10.7759/cureus.48757

**Published:** 2023-11-13

**Authors:** Andrew O Hess, Russell S Terry, Brian C Lobo, Jeb M Justice

**Affiliations:** 1 Otolaryngology, Dartmouth-Hitchcock Medical Center, Lebanon, USA; 2 Urology, University of Florida College of Medicine, Gainesville, USA; 3 Otolaryngology-Head and Neck Surgery, University of Florida College of Medicine, Gainesville, USA

**Keywords:** renal cell carcinoma (rcc), sinonasal metastasis, renal cell carcinoma metastasis, skull base tumors, clear cell renal carcinoma

## Abstract

Metastatic lesions to the paranasal sinuses and skull base, while rare, carry a poor prognosis. Renal cell carcinoma has been reported in multiple case reports to be one of the most common distant malignancies to spread to the paranasal sinuses; however, it is often unrecognized by physicians, and thus treatment is delayed. To increase awareness of this disease process, we describe three cases of metastatic renal cell carcinoma to the sinonasal cavity, which is the largest case series in the literature to date.

## Introduction

Renal cell carcinoma (RCC) has a relatively low incidence in the general population (approximately 17 cases per 100,000 population per year), but carries significant morbidity [1]. Up to 30% of patients have metastases at the time of initial diagnosis. The five-year relative survival for patients with metastatic disease is approximately 8-12% [2]. Even in patients with localized RCC managed surgically by either partial or radical nephrectomy with curative intent, up to 20% will recur within five years of treatment, and most of those recurrences will be distant metastases [3]. The most common sites of RCC metastases are the lungs (45%), bones (30%), lymph nodes (22%), liver (20%), adrenal (9%), and brain (8%) [4], but up to 20% of lesions can occur in other less common sites, including the sinonasal cavity (<1%) [5-7]. Delay in diagnosis for sinonasal RCC often occurs for several reasons: its rarity, delayed onset, and failure to include the sinonasal cavity in RCC imaging surveillance protocols. We describe three cases of metastatic RCC to the sinonasal cavity, two of which occurred six years after initial nephrectomy, with a goal of increasing clinicians’ awareness of this disease to include it in their differential diagnosis and look for it on pathology. 

## Case presentation

Case 1

**Figure 1:**
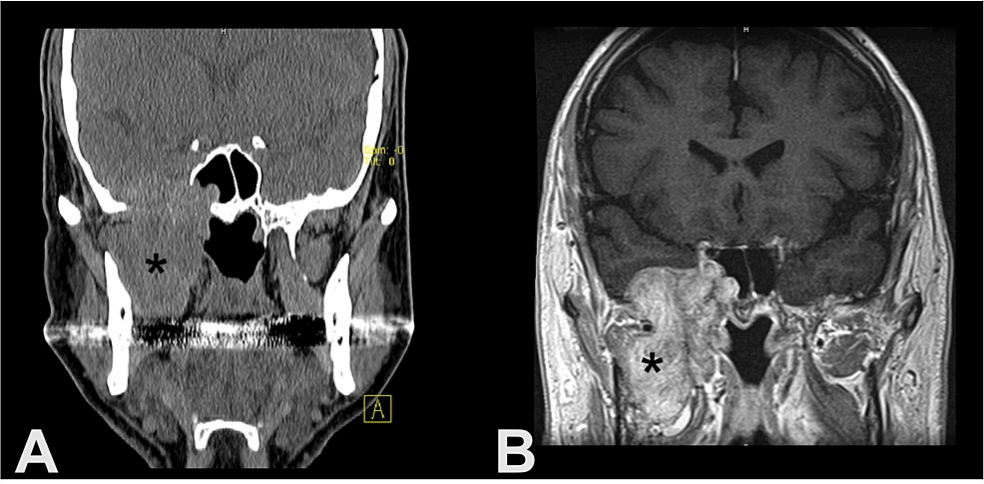
Image depicting RCC metastasis to the IT fossa, middle fossa, and sphenoid sinus (*) as seen on coronal CT of the head (A); image of RCC metastasis to the IT fossa, middle fossa, and sphenoid sinus (*) as seen on coronal MRI of the head (B). RCC: Renal cell carcinoma.

A 71-year-old man presented to an outside otolaryngologist with right unilateral hearing loss and aural fullness. His medical history was significant for type 2 diabetes, myelodysplastic syndrome, hypothyroidism, and right radical nephrectomy for clear cell RCC pathological stage T1b NX MX six years prior to presentation. The exam at that time showed right serous otitis media; however, nasal endoscopy did not note a nasopharyngeal mass. He subsequently had a myringotomy with pressure equalizing tube placement but continued to drain amber fluid. Four months later, he presented to our institution, and CT (Figure 1A) imaging was obtained, which showed a large right skull base mass involving the infratemporal fossa, eustachian tube, sphenoid sinus, cavernous sinus, and middle fossa dura. MRI (Figure 1B) was obtained, which confirmed a highly vascular mass, and nasal endoscopy did show subtle findings of hypervascularity in the lateral right nasopharynx and torus tubarius. He was taken for a biopsy, during which he lost a substantial 500 mL of blood, and metastatic renal cell carcinoma was confirmed. Full-body imaging showed additional lesions in both lungs as well as the left adrenal gland. Palliative radiation of 30 Gy in 3 Gy fractions was administered to slow the growth of these masses. Unfortunately, imaging at three months after radiation showed a continued lung mass growth of 5.4 mm over two years. Targeted chemotherapy was recommended at that time but refused by the patient. The patient developed profuse epistaxis eight months after a sinonasal biopsy, which was only controlled with embolization of the distal right internal maxillary artery. After discussion with the patient and family, the patient elected to forego aggressive treatment and lived another month under palliative care before passing away, approximately nine months from the date of presentation to our facility.

Case 2

**Figure 2:**
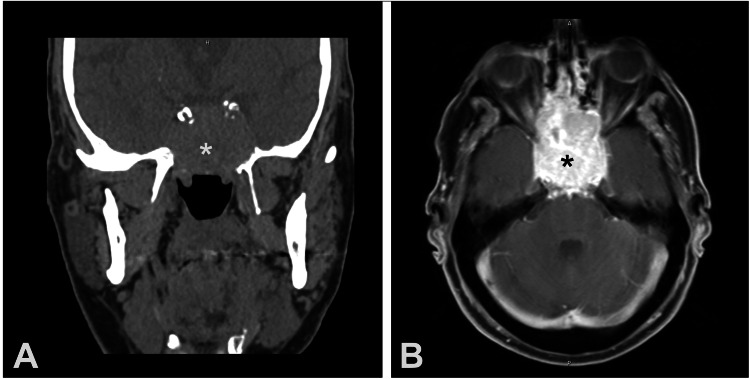
Image depicting RCC metastasis to the sphenoid, posterior ethmoids, sella, and clivus (*) as seen on coronal CT of head (A), image of RCC metastasis to the sphenoid, posterior ethmoids, sella, and clivus (*) as seen on axial MRI of head (B). RCC: Renal cell carcinoma.

A 79-year-old man presented to our emergency department with blurry vision, headache, and facial pressure for four months. His past medical history included hypertension, asthma, and a history of left radical nephrectomy for clear cell RCC six years prior with an unknown grade or stage. Head CT (Figure 2A) showed concern for a sinus mass, and dedicated maxillofacial CT again showed a large mass in the sphenoids, posterior ethmoids, sella turcica, and clivus. MRI (Figure 2B) confirmed a vascular lesion in the sphenoid, sella turcica, and clivus abutting the optic nerves and carotid arteries. At the time of presentation, the patient was in a hypertensive crisis with evidence of a mass on his right adrenal gland. However, pheochromocytoma was ruled out via laboratory studies. The patient’s blood pressure was normalized over a few days, after which he underwent transnasal endoscopic biopsies. A 500 mL blood loss was reported during the biopsy, and a confirmatory metastatic renal cell carcinoma diagnosis was established. The tumor board team recommended external beam proton radiation, which was completed; however, the patient passed away one month after this therapy under the care of a home hospice.

Case 3

**Figure 3:**
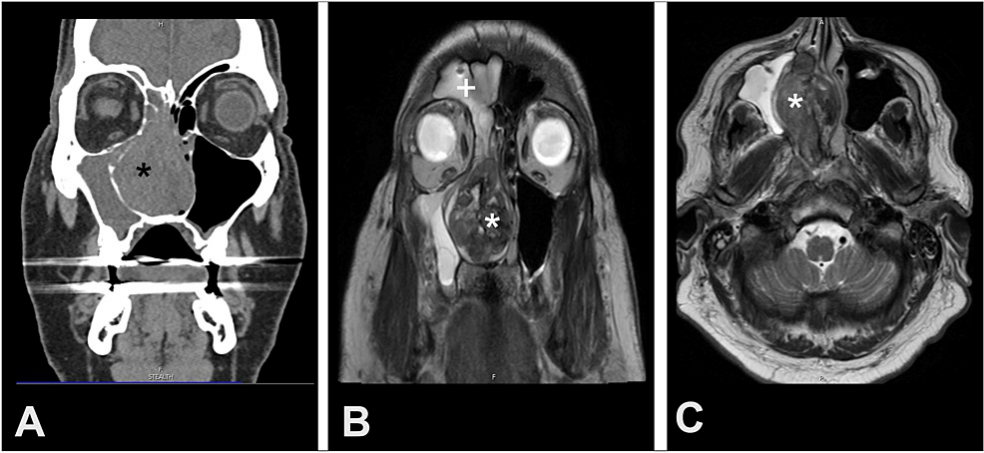
Image depicting sinus mass (*) as seen on coronal CT of head (A), image of sinus mass (*) and brain mass (+) as seen on coronal MRI of head (B), sinus mass (*) as seen on axial MRI of head (C).

A 73-year-old man presented to an outside otolaryngology clinic with a six-month history of right unilateral nasal obstruction and epistaxis. His only past medical history was hypertension and a history of right radical nephrectomy for clear cell RCC three years prior with an unknown grade or stage. Nasal endoscopy by the outside otolaryngologist showed a large vascular mass, and CT (Figure 3A) and MRI (Figures 3B, 3C) confirmed a large mass in the right nasal cavity, ethmoid sinus, and maxillary sinus. MRI also confirmed a lesion in the right temporal lobe of the brain. Polypectomy was scheduled to be performed but terminated early due to severe blood loss (750 mL). A biopsy was obtained during the procedure, which confirmed metastatic RCC. The patient was then referred to our facility for further management. Approximately six weeks after the initial biopsy, the patient underwent pre-operative embolization of the right distal internal maxillary artery with Onyx and subsequent endoscopic resection of the nasal mass with negative margins. Blood loss was 750 mL, but embolization did seem to help this case have less blood loss than the previous two cases. Full-body imaging for evidence of other metastases showed a 2-centimeter left adrenal mass. One week later, he underwent stereotactic radiosurgery of 20 Gy delivered in one fraction for the right brain lesion. The patient started medical treatment with axitinib 5 mg twice a day and pembrolizumab therapy every six weeks, along with scheduled imaging surveillance. At the time of this report, approximately five months have passed since tumor resection, the patient is tolerating treatment well, and metastatic disease is controlled.

## Discussion

RCC accounts for approximately 4% of new cancer cases and 2% of cancer deaths annually in the United States [1]. Over the past several decades, as the number of abdominal imaging studies performed for a variety of indications within the population has increased, so too has the incidence of RCC increased. This phenomenon has been accompanied by a downward-stage migration since tumors are often identified early while they are still localized, and surgical management is most often curative [8]. There is a male-to-female predominance of 1.75 to 1, and incidence generally increases with age. In our cohort, all patients were male and in their seventh or eighth decade of life at the time of their primary RCC diagnosis and treatment.

RCC is not a single neoplasm, but rather a histopathologically diverse group of several different neoplastic subtypes with varied etiology, biology, and prognoses [9]. The most common subtype, clear cell (70%), generally has the worst prognosis compared to the next two common subtypes: papillary (16.6%) and chromophobe RCC (5.9%) [10]. Nonetheless, five-year survival rates for most subtypes, including clear cell, can still exceed 90% with appropriate surgical management of a localized primary tumor at a low pathologic stage [11].

Metastatic rates for RCC are relatively high, with some of these metastatic lesions occurring even decades after surgical removal of the primary tumor [12]. RCC is the third most common malignancy to metastasize to the head and neck behind breast and lung cancer [12], with 15-30% of all metastatic RCC cases metastasizing to this region, most commonly to the parotid gland, thyroid, and larynx [13]. RCC metastases to the sinonasal cavity, however, are exceedingly rare [14]. Other malignancies have reports of sinonasal metastases, including lung, urogenital ridge, breast, gastrointestinal tract, and thyroid, but RCC is the dominant source, accounting for almost half of all cases, although it is extremely uncommon in all cases [15]. The clinical course of RCC is often unpredictable with regard to the timing of distant metastases. In a review of 7386 metastatic RCC cases, 15% of metastases were found two to seven years after primary diagnosis [16]. For some patients with metastatic RCC, symptoms of tumor metastasis can be the presenting sign that leads to their diagnosis. Approximately 7.5% of patients report head and neck symptoms as the initial complaint, which ultimately leads to their RCC diagnosis [13]. For each of our patients, metastatic lesions were found three to six years after the primary diagnosis, and all patients received a radical nephrectomy.

Metastases to the sinonasal cavity from a primary RCC are thought to occur through either hematogenous or lymphatic supply [17], but the exact mechanism remains unclear [6]. There are two potential hematogenous routes recorded. The first is the caval route where cells travel through the inferior vena cava, to the right heart, the lungs, the left heart, and up to the sinonasal region via the maxillary artery [18]. In the second route, metastatic cells can bypass pulmonary capillary filtration via Batson’s paravertebral venous plexus (extensive anastomoses between the avalvular vertebral and epidural venous system) and further travel to the intracranial and cavernous venous plexus to reach the nasal and paranasal sinuses [6,12,17-19]. The increased retroperitoneal pressure from the renal tumor shunts flow to the paravertebral plexus increasing the likelihood of spreading to the head and neck [12]. Spread via the lymphatic system is postulated to occur through the thoracic duct and then to the head and neck via retrograde flow through intercostal, mediastinal, or supraclavicular lymph vessels [6]. The maxillary sinus is the most common site of sinonasal metastases (36%), followed by the ethmoid sinus (25%), the frontal and sphenoid sinus (17%), and the nasal cavity (11%) [20]. Most sinonasal metastases are associated with synchronous metastasis to other regions [7]. This pattern was observed in our patients as simultaneous lesions were found in various regions of the body, including the adrenal gland, seen in two patients, the lung, and the right brain, although none of these were biopsy-proven RCC metastases. While the maxillary sinus is the most common location for metastasis in the sinonasal region, it was only involved in one of our patients. Two of our patients had sphenoid sinus involvement, which only made up 17% of cases in one review of 98 sinonasal metastases [20].

Sinonasal metastases have been reported as being the presenting sign of disease in a few cases [21,22], but more commonly are found at the time of the primary tumor diagnosis or decades later [23,24]. The most common presenting symptom of lesions in the sinonasal cavity is epistaxis (57.1%) due to the hypervascularity of these tumors, with other symptoms including facial swelling, facial numbness or pain, headache, epiphora, exophthalmos, diplopia, decreased vision and ptosis, and nasal obstruction depending on the size and location of the tumor [5,6,13,19,23]. RCC, particularly the common clear cell subtype, is classically associated with an inactivating mutation of the von Hippel-Lindau (VHL) gene, which results in significant upregulation of vascular endothelial growth factor A (VEGF-A). Although upregulation of VEGF-A1 is not unique to RCC, this dysregulation of angiogenesis leads to the classically hypervascular character of clear cell RCC tumors, and it is hypothesized to account for the possibility of blood loss when biopsied seen in patients who present with this problem [25,26]. In our patients, however, only one patient presented with epistaxis along with nasal obstruction on the ipsilateral side. The other two patients present with unilateral hearing loss with accompanying aural fullness and blurry vision, respectively. This may be explained by the fact that the patient with epistaxis had his lesion anterior in the anterior nasal cavity and was exposed to dryness from airflow, whereas the two patients without epistaxis had their masses deep in the skull base and sinuses. Although most literature does not report the duration of symptoms, our patients endured their head and neck symptoms for four to six months before presenting to our clinic. 

Diagnosing these metastatic tumors can be difficult due to the rarity of occurrence combined with the multitude of alternative diagnoses. A proper differential diagnosis should expand to include squamous cell carcinoma, adenocarcinoma, angiofibroma, inverted papilloma, hemangiopericytoma, melanoma, hemangioma, metastatic tumors from the breast and lungs, and systemic diseases such as Wegener’s and midline granuloma [19,23]. Paranasal sinus CT, contrasted CT, and MRI can help elucidate tumor characteristics such as tumor expansion, bone erosion, and remodeling (signs of malignant and metastatic lesions) as well as vascularity [19]. Biopsy of the mass is required for a final diagnosis and should be guided by contrasted CT to avoid uncontrolled hemorrhage from these highly vascular tumors [6,13]. For patients with a known history of RCC, selective embolization of the tumor prior to biopsy is recommended by some authors [19]. Each of our patients received both non-contrast CT and confirmatory MRI, which elucidated the sinonasal involvement as well as several characteristics, such as the vascularity of the lesions. It is important to note that the first patient did receive a nasal endoscopy, which did not display signs of a mass, and therefore imaging is required for the early detection of such lesions. For each of our patients, pathology results from biopsies confirmed clear-cell RCC. Blood loss during biopsy was 500 mL or greater in all cases, further confirming the increased vascular density of these lesions. 

The overall five-year survival rate for metastatic RCC is approximately 8-12%. Five-year survival rates can improve to 13-35% for patients after the removal of the primary tumor, regardless of the time interval between nephrectomy and the time of metastatic lesion diagnosis [27,28]. Treatment for metastatic RCC typically follows the National Comprehensive Cancer Network (NCCN) guidelines, which include resection of the primary tumor and metastasectomy or stereotactic body radiation therapy. A tailored regimen of chemotherapy such as axitinib and pembrolizumab is also recommended [29]. Significant advancements are currently being made in the development and testing of new immunotherapy agents, such as anti-programmed cell death 1 (PD-1) inhibitors, for these cases [30]. Surgical metastasectomy continues to be the best long-term survival option, especially for single metastatic tumors. Resection of the primary tumor and metastatic lesions renders a 41% survival in two years and 13% in five years. Patients with multiple metastatic masses can expect a much lower survival rate of 0-7% in five years [23]. Only one of our patients received surgical removal of the sinonasal mass. Consistent with most literature, this patient has had the best prognosis when compared to the other two patients. For patients who are poor candidates for surgery, radiotherapy, immunotherapy, and chemotherapy are available; however, metastatic RCC has a relatively poor response to some of these modalities [6,13]. While primary RCC is typically radio-resistant because of the surrounding tissues, metastatic lesions can respond well to higher doses of therapy [24]. In our cohort, only two patients received radiation, with varying results. For the first patient, radiotherapy did not significantly inhibit the growth of the tumors, and he died eight months after radiotherapy (nine months from the initial metastatic diagnosis). The second patient did receive a full course of proton beam radiation, but ultimately succumbed to his disease two months after radiotherapy (six months from the initial metastatic diagnosis). The third patient underwent stereotactic radiosurgery; however, it was not targeted at his sinonasal lesion but rather the metastatic disease in the brain. Our third patient is also currently receiving axitinib and pembrolizumab as immunotherapy with palliative intent for approximately three months and regular image surveillance for potential metastatic lesions.

## Conclusions

The aggressive nature of metastatic RCC elicits a poor prognosis, necessitating early diagnosis and treatment whenever possible. Early diagnosis of sinonasal metastases is paramount as these lesions are typically resistant to chemotherapy and radiation; thus, diagnosing the lesion before it is too large is important if surgery is the only option. The purpose of this case series is to illustrate the presenting symptoms of the rare form of sinonasal metastasis to aid in the early diagnosis and treatment of these patients. Any patient with a history of RCC and a new sinonasal or skull base mass should have metastatic RCC in the differential, even if over five years since the initial diagnosis.
